# Second-Language Acquisition and First-Language Attrition of Speech: The Production of Arabic and English Short Vowels

**DOI:** 10.1177/00238309251344889

**Published:** 2025-08-10

**Authors:** Lisa Kornder, Amirah Saud Alharbi, Anouschka Foltz

**Affiliations:** University of Graz, Austria; Umm Al-Qura University, Saudi Arabia; University of Graz, Austria

**Keywords:** Vowels, English, Arabic, first-language attrition, second-language acquisition

## Abstract

This study investigates if two groups of experienced late bilinguals (Arabic-English, English-Arabic) produce the Arabic vowels /ɪ, u, a/ and the English vowels /ɪ, ʊ, æ/ with nativelike formant values (F1, F2) compared with Arabic and English monolinguals, respectively. We aimed to characterize the relationship between second-language (L2) acquisition and first-language (L1) attrition of vowels, that is, does nativelike acquisition of an L2 vowel correspond to attrition of a phonetically similar L1 vowel, and vice versa? Moreover, we explored if nativelikeness of bilingual vowel productions is influenced by the predictor variable sound discrimination aptitude. Results show that bilinguals who produce nativelike L2 vowels are also able to maintain native L1 productions, suggesting that an increased L2 proficiency does not inevitably entail a decline in L1 proficiency.

## 1 Introduction

Many vowel production studies have shown that late L2 learners, despite prolonged L2-learning experience, often fail to produce L2 vowels with nativelike acoustic features (e.g., Baker & Trofimovich, 2005; Bohn & Flege, 1992; Cobb & Simonet, 2015; Flege et al., 1997, 1999, 2003; Levy & Law, 2010; Piske et al., 2002). Within the framework of the Speech Learning Model (SLM; Flege, 1995; see Flege & Bohn, 2021, for a revised version of the SLM), category *assimilation* is one of the mechanisms assumed to underlie this inability, such that L2 learners fail to discern acoustically similar L1 and L2 sounds, resulting in a merged L1/L2 category that is specific to neither the L1 nor the L2 (e.g., Flege, 2002). Alternatively, category *dissimilation* may occur, mostly among, but not restricted to, early bilinguals (e.g., Flege et al., 2003; Guion, 2003; Lee & Iverson, 2012; Oh et al., 2011). This mechanism leads to increased phonetic contrast between similar L1 and L2 sounds, that is, the related sounds move further apart, resulting in nonnative phonetic categories (Flege, 1995; Flege & Bohn, 2021).

Assimilation and dissimilation processes may not only impact L2 productions but have also been shown to affect L1 phonetic features; that is, bilinguals may produce sounds (e.g., Alharbi et al., 2023; Bergmann et al., 2016; Kornder & Mennen, 2021; Mayr et al., 2012) or prosodic features (e.g., De Leeuw et al., 2012; Mennen et al., 2022) in their L1 differently from monolinguals. Such L2-induced modifications of L1 pronunciation experienced by L2-immersed late bilinguals are referred to as *L1 phonetic attrition* (Köpke & Schmid, 2004).^1^

In terms of vowel production, research has shown that the presence of an L2 system might lead to changes in the realization of L1 vowels, that is, a bilingual’s L1 vowel categories may move acoustically closer (as evidenced in, for example, formant frequency changes) to corresponding L2 vowels, or similar L1 and L2 vowels move further apart (e.g., Bergmann et al., 2016; Flege, 1987; Kornder & Mennen, 2021; Mayr et al., 2012).

While L2 acquisition and L1 attrition of segments are well-studied phenomena, there is still a small number of studies that explore how acquisition and attrition are *related* by comparing L1 and L2 speech production (see Alharbi et al., 2023; Kornder et al., 2023; Kornder & Mennen, 2021; Mayr et al., 2012; Mennen, 2004; Stoehr et al., 2017, for exceptions). Is, for example, an advanced L2 pronunciation proficiency accompanied by a reduction of proficiency in the L1, and vice versa? That is, do native-like L2 realizations entail nonnative realizations of the corresponding features in the L1 (see, for example, Flege, 2003)? To broaden our understanding of the acquisition-attrition-relationship, the present study investigated L1 and L2 vowel productions in two groups of advanced late bilinguals, namely, Arabic-English (A-E) and English-Arabic (E-A) bilinguals who have been long-term immersed in an L2-country (Great Britain or Saudi Arabia). The research presented in this paper is part of a larger study that aimed to explore segmental and prosodic production in bilinguals’ first (Arabic or English) and second (English or Arabic) language. We assessed the bilinguals’ speech production by acoustically investigating their realization of voice onset time in word-initial plosives in Arabic and English (reported in Alharbi et al., 2023), their production of Arabic and English short vowels, as discussed in the present paper, as well as their production of Arabic and English long vowels and their realization of prosodic patterns in *wh*-questions (both in preparation). It is one of the few production studies that looks at both acquisition and attrition in bilingual speakers with the same combination of languages, but who differ with regard to the order of acquisition of these languages. Not only comparing bilingual productions to monolingual productions but also comparing the two bilingual groups with each other is particularly relevant given the well-accepted observation that “L2 learners can never perfectly match monolingual native speakers of the target L2” (Flege & Bohn, 2021, p. 64) due to the fact that a bilingual’s linguistic systems constantly interact with each other.

### 1.1 Arabic and English vowels

The target varieties in our study are Modern Standard Arabic (MSA) and Standard Southern British English (SSBE), which are representative of the standard-oriented varieties spoken by our participants.

SSBE distinguishes six short monophthongs /ɪ, ɛ, a, ʌ, ɒ, ʊ/, five long monophthongs /iː, ɜː, ɑː, ɔː, uː/, and eight diphthongs /eɪ, aɪ, ɔɪ, ǝʊ, aʊ, ɪǝ, eǝ, ʊǝ/ that can occur in stressed syllables. English vowels can be characterized in terms of vowel quality (vowel height and vowel frontness) and duration, with the latter distinguishing English short from long monophthongs (e.g., Ladefoged, 2005; Williams & Escudero, 2014).

Compared with English, MSA has a simpler vowel system with only three short /ɪ, a, u/ and three long /iː, aː, uː/ monophthongs as well as two diphthongs /aw, aj/ (some Arabic dialects have more distinctions though; see, for example, Holes, 2004). MSA short vowels contrast with their long counterparts, that is, vowel length is phonemic in Arabic (e.g., Alghamdi, 1998; De Jong & Zawaydeh, 2002; Ryding, 2005), for example, /dʒaˈm**a**l/ جَمل “camel” vs. /dʒaˈm**aː**l/ جَمال “beauty.” There are, however, also differences in vowel quality, such that Arabic long vowels are overall more peripheral compared with their short, more centralized counterparts (e.g., Alghamdi, 1998).

Here, we focus on the short monophthongs that are phonetically similar but not identical (i.e., they differ acoustically in terms of F1 and F2; see section 3.1) in Arabic and English, namely, short /a-æ/, /ɪ/ and /u-ʊ/.^2^

### 1.2 Individual differences

Individuals may differ from each other in terms of successful L2 acquisition and L1 maintenance. Such differences are evident even among age-matched bilinguals with similar language learning histories, and they may be related to various cognitive, motivational and attitudinal factors (see Hansen Edwards, 2018, for an overview). In our study, we explore if and to what extent *sound discrimination aptitude* may or may not influence vowel production accuracy in our bilingual speakers.

#### 1.2.1 Sound discrimination aptitude

Sound discrimination aptitude refers to a cognitive ability that is largely outside one’s control. This capacity is commonly considered an integral part of general language learning aptitude, that is, the innate *ability* or *talent* to learn languages (Carroll, 1981). Sound discrimination aptitude reflects one subcomponent of aptitude introduced by Carroll (1971), namely, *phonemic coding ability*, that is, “the ability to identify and store in long-term memory, new language sounds or strings of sounds” (Carroll, 1971, p. 4). When it comes to general language learning aptitude, the assumption is that the acquisition of different aspects of language (pronunciation, grammar, lexicon, etc.) requires different cognitive skills, such that, for example, the (successful) acquisition of pronunciation requires enhanced phonemic coding ability as part of overall language learning aptitude (see Baker Smemoe & Haslam, 2013).

In the context of bilingual speech acquisition, the ability to perceive distinct phonetic characteristics of L2 sounds and distinguish them from perceptually-linked L1 sounds is considered essential for accurate production (e.g., Flege, 1995), though more recent models on L2 speech acquisition propose that the link between speech sound perception and production abilities is less straightforward than that (see De Leeuw & Chang, 2024; Flege & Bohn, 2021). As phonemic coding ability includes both sound discrimination and mimicry abilities, individuals with a low phonemic coding ability may encounter difficulties when identifying and remembering unfamiliar sounds and are also less likely to produce them accurately (e.g., Flege et al., 1999; Purcell & Suter, 1980; Saito et al., 2019; Thompson, 1991).

In the context of L1 attrition, speech *perception* studies have assessed bilinguals’ abilities to accurately perceive L1 sounds (e.g., Cabrelli et al., 2019; Dmitrieva, 2019; Kellogg & Chang, 2023). These studies suggest that the way bilinguals perceive or distinguish L1 sounds may be modified as a result of L2 learning experience. That is, they may experience difficulty in accurately perceiving sounds or sound contrasts in their native language. In our study, by contrast, we did not set out to test bilinguals’ ability to discriminate speech sounds in their L1/L2, that is, we did not assess their perception *proficiency* (as has been done in the studies cited above), but we aimed to find out if the cognitive mechanism of sound discrimination aptitude is likely to influence our bilinguals’ production accuracy. As discussed in section 2, sound discrimination aptitude is typically investigated in tests that use a language participants are not familiar with (in our study, we used Cantonese). Using an unknown language allows gaining insights into participants’ aptitude by excluding proficiency in a (known) language as a potentially confounding factor. Hence, in our study, we explored if increased sound discrimination aptitude may be related to more nativelike L1/L2 vowel productions in our bilingual speaker groups.

### 1.3. Research questions

The present study aims to expand on the body of research exploring the relationship between L2 acquisition and L1 attrition of *segmental speech production*. We also aim to assess the impact of the predictor variable sound discrimination aptitude. We address the following research questions for Arabic and English vowels produced in isolation and in a carrier phrase:

**RQ1:** To what extent are highly-proficient Arabic-English and English-Arabic bilinguals able to produce their respective L2 vowels in a nativelike manner?
**RQ2:** Do these bilingual speakers show evidence of attrition in their respective L1 vowels?
**RQ3:** Can potential differences in bilingual speakers’ L1 and L2 vowels be related to differences in participants’ sound discrimination aptitude?
**RQ4:** Do nativelike realizations of L2 vowels entail nonnative-like productions of the corresponding L1 vowel targets and vice versa?

## 2 Methods

### 2.1 Participants

The study included four speaker groups (*n* = 15 each): One group comprised Arabic-English bilinguals (1 male, 14 females^3^; mean age = 39.4 years; range = 35–50 years; *SD* = 3.76) who had moved to the United Kingdom (Sheffield, Chester or London) from Saudi Arabia (Jeddah, Makkah or Riyadh) or from eastern Yamani in early adulthood (mean age of arrival, AoA = 18.6 years; *SD* = 1.72). They reported a mean length of residence (LoR) in the L2 country of 20.27 years (*SD* = 1.87). The Arabic-English bilinguals were native speakers of Modern Standard Arabic (MSA), who used MSA on a regular basis in professional contexts and completed the Arabic tasks also in MSA.^4^ As their L2, they had acquired Standard Southern British English (SSBE).

The second bilingual group included English-Arabic bilinguals (4 males, 11 females; mean age = 33.93 years; range = 33–39 years; *SD* = 1.58) from the United Kingdom (Sheffield or London) who had moved to Saudi Arabia or Yemen in late adolescence (mean AoA = 16.73; *SD* = 1.44), with a mean LoR of 16.93 (*SD* = 1.03). The English-Arabic bilinguals spoke SSBE as their L1, perhaps with some regional influences, depending on the region they came from. They were fluent speakers of L2 MSA and, as the Arabic-English bilinguals, completed all Arabic tasks in MSA.

Participants in both bilingual groups had fully acquired their L1 (Arabic or English) in their country of birth and had learned their L2 (English or Arabic) in late adolescence or early adulthood, that is, they can be described as late consecutive bilinguals. Based on the results of the grammar and listening comprehension parts of an English proficiency test (a practice version of the Test of English as a Foreign Language; Educational Testing Services [ETS], 2021) (*M* = 42.1; *SD* = 1.8; range = 39–45, possible maximum: 45) and an Arabic proficiency test^5^ (*M* = 43.6; *SD* = 1.5; range = 42–46, possible maximum: 46), the bilinguals in the present study were considered to be highly proficient speakers of their respective L2.

Monolingual Arabic and monolingual English speakers served as controls and were matched to the bilingual speakers as closely as possible in terms of level of education (all participants held at least a Bachelor’s degree), regions of residence and thus regional dialects, as specified above. The Arabic monolinguals (4 males, 11 females; mean age = 36.07; range = 30–56 years; *SD* = 7.20) lived in different regions in Saudi Arabia (Jeddah, Makkah, Riyad, and Abha) and the English monolinguals (2 males, 13 females; mean age = 39.87; range = 32–60 years; *SD* = 9.06) in the United Kingdom (Sheffield, Chester, and London) at the time the study was conducted. None of the monolingual participants reported knowledge of additional languages. All participants provided written informed consent for us to conduct and publish the study prior to participating.

All four participant groups were matched as closely as possible in terms of age and gender. A linear model with age as response variable and participant group as fixed factor (sum-coded for ANOVA-style main effects) shows that the four participant groups do not differ significantly in age (β = -0.15, *SE* = 0.84, *t* = -0.18, *p* = .859). A generalized linear model for count data and contingency tables (Poisson regression) with counts of female participants as response variable and participant group as fixed factor (sum-coded for ANOVA-style main effects) shows that the four participant groups do not differ significantly in terms of gender distribution (β = -0.03, *SE* = 0.17, *z* = -0.19, *p* = .848). The two bilingual participant groups were also matched as closely as possible in terms of AoA and LoR. Two linear models with participant group as fixed factor (sum-coded for ANOVA-style main effects), one with AoA and one with LoR as response variable, suggest that the two bilingual groups do differ significantly both in terms of AoA (β = -0.95, *SE* = 0.29, *t* = -3.22, *p* = .003) and LoR (β = -1.70, *SE* = 0.28, *t* = -6.04, *p* < .001). Even though the two groups only differ in mean AoA and mean LoR by 2 and 3 years, respectively, these relatively small differences are significant.

### 2.2 Materials

All anonymized data, analysis scripts, and a result summary are available at https://osf.io/sp2mq/.

#### 2.2.1 Vowel productions

We obtained Arabic and English vowel production data for the short vowels /a-æ/, /ɪ/ and /u-ʊ/ through a reading task. Some studies suggest that L1 attrition effects may be more pronounced in casual speaking styles (spontaneous conversation) than in formal contexts (sentence or word-list reading) because more formal speaking contexts allow participants to pay closer attention to their pronunciation (e.g., Hévrová et al., 2020; Major, 1992). Our aim, however, was to include an equal number of vowel repetitions produced by the individual speakers to provide a representative comparison of their L1 and L2 productions across and within speaker groups. As discussed in previous studies on L1 attrition and L2 acquisition of segmental speech production, using (semi-)spontaneous speech samples does not always allow for testing a balanced number of tokens per target sound and speaker (see Bergmann et al., 2016; Kornder & Mennen, 2021). In addition, as vowels are particularly prone to be affected by co-articulation, we wanted to ensure that the target vowels occurred in a neutral /hVd/ consonant framework in both languages (see, for example, Di Paolo et al., 2011; Robb & Chen, 2009), which would have been rather difficult to control for—if not impossible—in spontaneous speech samples. Accordingly, the English target words included in our study were *hid* /hɪd/, *had* /hæd/, and *hood* /hʊd/, eliciting the vowels /ɪ/, /æ/ and /ʊ/, respectively. The Arabic targets included both words and nonwords (indicated through *) and were هِد* /hɪd/, هَد /had/ (English: *demolish*), and هُد*/hud/, eliciting the vowels /ɪ/, /a/ and /u/, respectively.

Following Mayr et al. (2012), we asked our participants to produce the target words both in isolation and in a fixed carrier phrase. Even though we do not expect considerable production difference in these two conditions, vowels produced in an isolated context may be slightly more peripheral than vowels produced in an embedded context. In our analyses, we do not collapse these two conditions but include speaking condition as a factor (see section 2.2.6) to be able to compare our findings to previous vowel production studies that typically make use of either isolated (e.g., Cobb & Simonet, 2015) or embedded (e.g., Levy & Law, 2010) elicitation tasks. To activate the intended vowel categories (particularly for Arabic nonwords), we primed the target words with real words containing the same vowel sound, for example, *would, could, hood* (see Appendix A).

#### 2.2.2 Language background questionnaire

We collected bilinguals’ demographic data (age, gender, years of L1/L2 instruction, language qualifications) using a Language Background Questionnaire (adapted from Schmid, 2002). We also asked participants to report on how frequently they used their respective L1 and L2 with their parents, siblings, partner/spouse, children, and others (including work contexts and friends; see Appendices B and C, for language use information).

#### 2.2.3 Sound discrimination

To investigate sound discrimination aptitude, bilinguals completed an adapted version of *Part V—Sound Discrimination* of the *Pimsleur Language Aptitude Battery* (PLAB; Pimsleur, 1966). The PLAB assesses foreign language learning aptitude by measuring various cognitive abilities considered to be relevant for language learning. These include verbal abilities, motivational factors and, in Part V, participants’ ability to learn and recognize new phonetic distinctions. We designed our test items using freely available online samples as a template (available at Language Learning and Testing Foundation [LLTF], 2019, online), that is, we created questions analogous to the sample questions. The PLAB Sound Discrimination test requires participants to identify and distinguish pitch, orality and nasality in a language that they are not familiar with. This allows tapping into individuals’ language aptitude (i.e., sound discrimination) without proficiency in a particular language as a confound. We used Cantonese as the test language since we had contact to a native Cantonese speaker with a high proficiency in English. During the test, participants learned three Cantonese words they were presented with in written and in audio form, including an English translation. We then tested their ability to recognize the newly learned words in a total of 30 spoken utterances, which each contained one of the three newly learned words.

#### 2.2.4 Procedure

We collected data in Saudi Arabia and in the United Kingdom, recruiting participants mainly via personal contacts. The bilingual groups participated in three sessions, each taking place on a different day, with a maximum of 1 week in between: In the first session, the bilinguals completed the proficiency test for their respective L2 and the sound discrimination task (see Alharbi, 2020, for additional tasks not considered here). We then collected Arabic and English production data in two separate sessions. For the vowel production task (see Alharbi et al., 2023; Alharbi, 2020, for plosive and prosody production tasks), participants read out the Arabic and English target words 3 times isolation and 3 times embedded in a carrier phrase while being recorded with a handheld Sony tape recorder (sony icp px333 Digital Voice Recorder). Monolinguals participated in a single session as they only completed the production tasks in their L1 and provided demographic information. The reading task resulted in a total of 1,608 vowel productions (12 productions are missing due to experimenter error), *n* = 798 for Arabic and *n* = 810 for English.

#### 2.2.5 Data pre-processing

We examined English and Arabic vowels by conducting formant analyses of F1 and F2, corresponding to vowel height and vowel frontness, respectively. Vowel height is negatively correlated with F1, that is, high vowels typically have low F1 values and low values have comparatively higher F1 values. Vowel frontness, by contrast, is correlated with F2, that is, front vowels have high F2 values while back vowels have comparatively lower F2 values (see, for example, Harrington & Cassidy, 1999). For each of the elicited vowels, we set segment boundaries manually in Praat (Boersma & Weenink, 2016) by identifying vowel onset and end point in the spectrogram. Formants were measured at the temporal mid-point of the vowel,^6^ using the *To Formant (burg)* method (Childers, 1978), with standard settings, that is, five calculated formants in the frequency range up to 5000 and 5500 Hz for males and females, respectively, an analysis-window length of 25 ms, and a pre-emphasis of 50 Hz. The resulting formants were corrected manually if tracking errors occurred.

To eliminate variation caused by physiological and age-related differences among female and male speakers, we normalized the formant values with the Bark Difference Method (modified from Syrdal & Gopal, 1986), using the *normBark* function in the phonR package (McCloy, 2016) in R (R Core Team, 2023). This method computes differences between Bark-converted values (Z). We obtained two measures, namely, Bark-converted F1 (Z3 minus Z1, which estimates vowel *height*, that is, higher Z3–Z1 corresponds to a higher tongue position) and Bark-converted F2 (Z3 minus Z2, which estimates vowel *frontness*, that is, higher Z3–Z2 corresponds to a less fronted tongue position). In the following, these will be referred to as F1-Bark and F2-Bark, respectively.

#### 2.2.6 Data analysis

For each vowel, we conducted three sets of analyses in R (R Core Team, 2023), with two analyses per set, one for tongue height (F1-Bark) and one for tongue frontness (F2-Bark). We decided to analyze the three vowels separately considering that our analyses would get too complex and difficult to interpret if we were to conduct one large analysis for all vowels. In addition, individual analyses allow us to more closely determine if and to what extent the vowels are affected differently by both acquisition and attrition. To see if the experimental manipulations affected L2 (RQ1) and L1 (RQ2) productions, we ran linear mixed-effects models using the lme4 package (Bates et al., 2015). The statistical model for each analysis included *language* (Arabic, English), *speaker group* (monolingual, Arabic-English bilingual, English-Arabic bilingual), *speaking condition* (isolated, sentence) and all interactions as fixed effects, random intercepts for *participants*, and random slopes for each participant for the within-participant factor *speaking condition*.

To see if participants’ individual differences in terms of sound discrimination aptitude affected vowel productions (RQ3), we ran further linear mixed-effects models restricted to the bilingual groups. The response variables for the models were the distance in tongue height (F1-Bark) and tongue frontness (F2-Bark), respectively, from the monolingual norms (distance-from-norm). To calculate these distances for each language, we subtracted the average F1-Bark and F2-Bark of the respective monolingual groups from each F1-Bark and F2-Bark value for bilingual participants and then took the absolute value of each difference. For example, if a bilingual participants’ F1-Bark value for a certain production of the English vowel /æ/ is 6.3 Bark and the average F1-Bark from monolingual productions of the English /æ/ is 8.3 Bark, then the distance in tongue height from the monolingual norm for this production is |6.3 – 8.3| = |−2| = 2 Bark. The larger this absolute distance from the respective norm, the less nativelike we consider participants’ productions to be. The statistical model for each analysis included *language* (Arabic, English), *speaker group* (Arabic-English bilingual, English-Arabic bilingual), *sound discrimination aptitude* (numeric), and all interactions as fixed effects. The models also included random intercepts for *participants*, and random slopes for each participant for the within-participant factors *language* and *sound discrimination aptitude* all well as their interaction.

For all linear mixed-effects models, we centered and sum-coded all fixed factors prior to analysis to avoid multicollinearity and to get ANOVA-style main effects and interactions. For each analysis, we then conducted model comparisons using the *anova()* function, which tests if the compared models differ statistically significantly in fit. If two models did not differ statistically significantly in fit, we chose the simpler model; otherwise, we chose the more complex one. We first simplified the random effects-structure, starting with the highest order effects and the smallest variance. We then simplified the fixed effects structure by removing nonsignificant effects, starting with the highest order effects and the highest p-value. We report the final models from these model comparisons throughout. If post hoc comparisons were needed, we calculated simple contrasts using the *emmeans()* function (Lenth, 2020). All linear mixed model results are presented in tables with significant p-values shown in bold face.

To see if successful L2 acquisition might entail L1 attrition (RQ4), we ran Pearson correlations for the bilingual participants.^7^ For each participant, vowel, speaking condition, and language, we calculated the distance in tongue height (F1-Bark) and tongue frontness (F2-Bark), respectively, from the monolingual norms. Unlike the previous analysis, where we calculated the distance of *individual* productions from the respective monolingual norms, here we took the average distance from the monolingual norm of all three productions that participants produced for each vowel, speaking condition, and language. For example, if a participant’s distances from the monolingual norm in terms of tongue height were 2 Bark, 1.5 Bark and 1.3 Bark, then we calculated the arithmetic mean of these three values, that is, 1.6 Bark. We then ran Pearson correlations separately for tongue height (F1-Bark) and tongue frontness (F2-Bark) to see if bilingual participants’ absolute distances from the Arabic and English norms correlate.

## 3 Results

### 3.1 Comparison with previous vowel studies

We first compare the F1/F2 vowel space for /a-æ/, /ɪ/ and /u-ʊ/ of our bilingual and monolingual speaker groups with published reference values (which are only available in Hertz for Arabic) to determine if the formant measurements obtained here are comparable to previous measurements. For Arabic, we chose the average F1 and F2 values of five male^8^ Saudi Arabic speakers reported in Alghamdi (1998). For English, we chose the weighted average^9^ F1 and F2 values of the male and female speakers in Deterding (1997). Raw and Bark-converted F1 and F2 values for each vowel, language, and speaking condition can be found in Appendices D to F.

**Figure 1.**
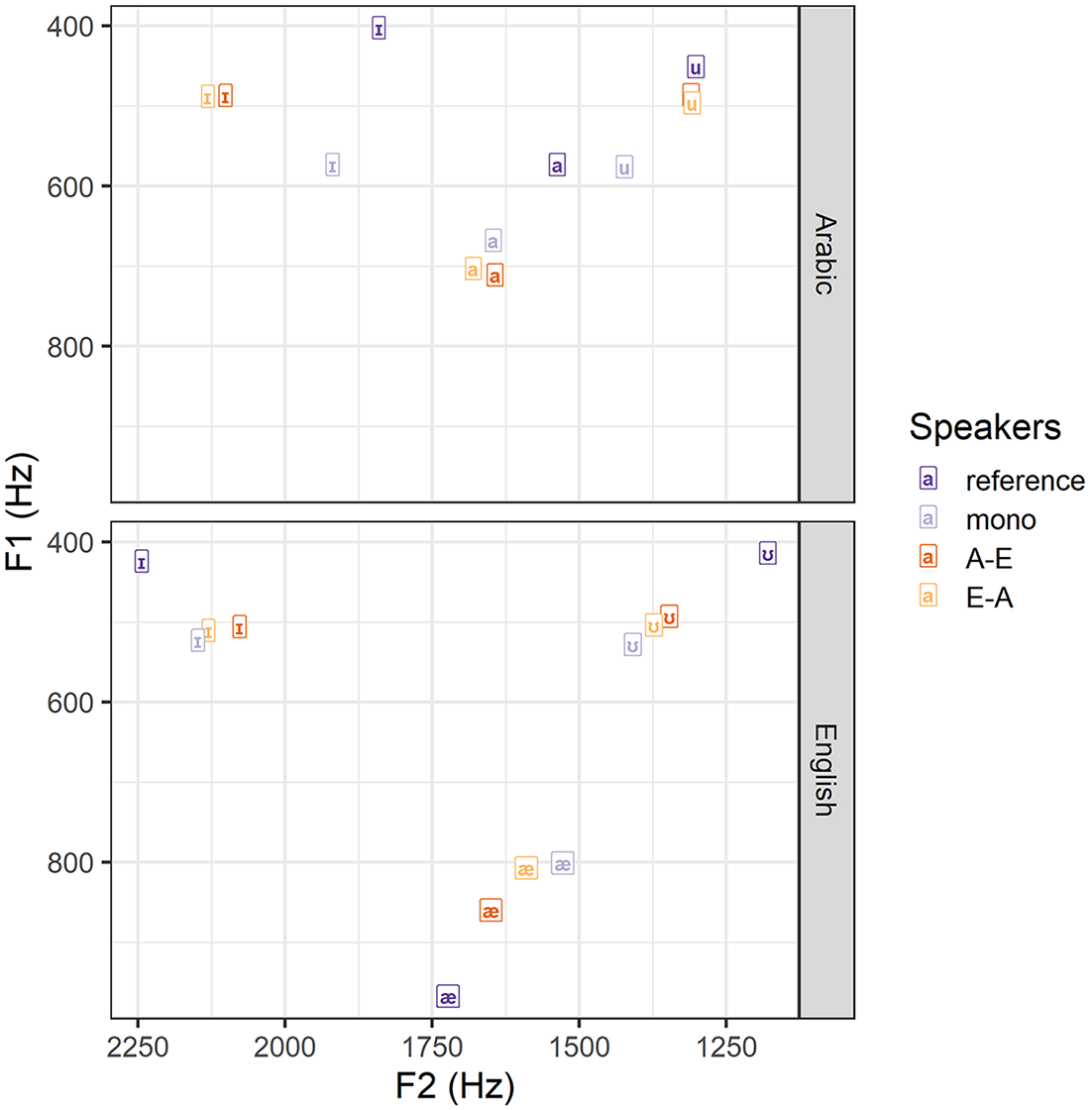
Vowel space for the Arabic and English short vowels /a/-/æ/, /ɪ/ and /u/-/ʊ/ for monolinguals, A-E bilinguals, E-A bilinguals, and reference values.

Figure 1 shows that our speakers’ Arabic F1 and F2 values are overall higher than the values obtained for Alghamdi’s (1998) Arabic speakers, most likely because Alghamdi (1998) included male speakers only, who tend to have lower F1 and F2 values compared with women (e.g., Huber et al., 1999; Traunmüller, 1988), while participants in our study were mainly female. Figure 1 also shows that our speakers’ English F1 and F2 values are less peripheral than those reported in Deterding (1997). This would be expected as our values comprise vowels from words produced in isolation and in a sentence, whereas values from Deterding (1997) come from citation forms. Overall, our F1 and F2 values for all three Arabic and English vowels are comparable to the values reported in previous studies.

### 3.2 Results of formant analyses

For each vowel we now analyze whether (a) the *experimental manipulations* language, speaker group and/or speaking condition affect F1-Bark and F2-Bark values, (b) bilingual participants’ *individual differences* in sound discrimination aptitude affect how nativelike their F1-Bark and F2-Bark values are, and (c) we find an *L2 acquisition, L1 attrition relationship*.

#### 3.2.1 Vowel /a/-/æ/

##### Experimental manipulations

**Figure 2.**
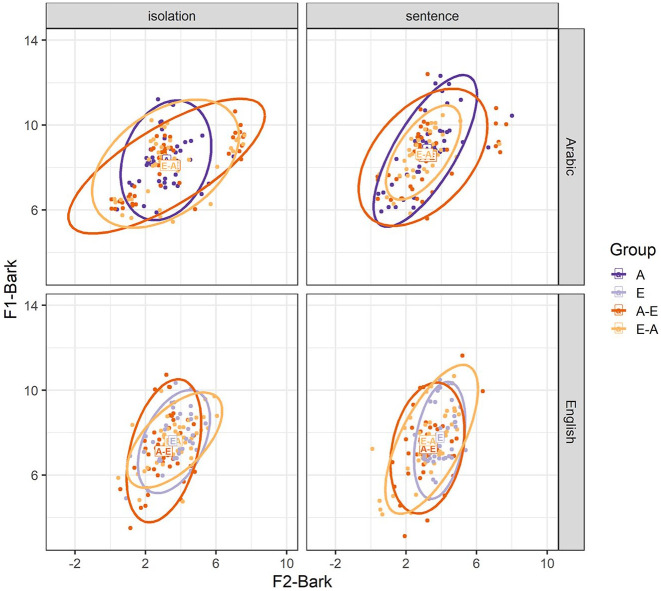
F1-Bark and F2-Bark values for Arabic /a/ and English /æ/ in the isolation and sentence conditions. The rectangles represent average F1-Bark and F2-Bark values for the Arabic monolinguals (A), English monolinguals (E), Arabic-English bilinguals (A-E), and English-Arabic bilinguals (E-A). Dots and ellipses show individual data points and distributions, respectively, for each participant group.

We first consider whether the experimental manipulations affect F1-Bark and F2-Bark values of the vowel /a-æ/. Figure 2 shows that Arabic /a/ is produced at an overall higher position, as manifested in higher F1-Bark values, compared with English /æ/. On the front-back axis, Arabic /a/ shows a larger spread in values compared with English /æ/.

The final statistical model for F1-Bark (fixed effects: language, speaking condition; random intercepts: participants; random slopes: speaking condition by participant) in Table 1 shows significant main effects of language, with significantly higher F1-Bark for Arabic compared with English, and speaking condition, with significantly higher F1-Bark for the sentence condition compared with the isolation condition.

**Table 1.** Statistical results for the experimental manipulations analysis for F1-Bark for the vowel /a/-/æ/.

final statistical model				
*effect*	*β*	*SE*	*t*	*p*
language	-0.46	0.06	-8.04	**<** 0.001^***^
speaking condition	0.15	0.07	2.27	**=** 0.027^*^

* *p* < 0.05; ***p < 0.001

The final model for F2-Bark had no fixed effects (random intercepts: participants), such that our experimental manipulations did not significantly affect tongue frontness.

##### Individual differences

We now explore if our individual differences measure affects the nativeness of /a-æ/ productions. The final model for the F1-Bark distance-from-norm analysis had no fixed effects (random intercepts: participants; random slopes: language by participant), such that speaker group, language, and sound discrimination aptitude did not affect /a-æ/ nativeness in terms of tongue height.

The final model for the F2-Bark distance-from-norm analysis (fixed effects: language; random intercepts: participants; random slopes: language by participant) in Table 2 shows a significant main effect of language, with significantly more target-like productions in terms of tongue frontness for English /æ/ than Arabic /a/. Sound discrimination aptitude did not affect the nativeness of participants’ /a-æ/ productions.

**Table 2.** Statistical results for the individual differences analysis for F2-Bark for the vowel /a/-/æ/.

final statistical model				
*effect*	*β*	*SE*	*t*	*p*
language	-0.17	0.08	-2.20	**=** 0.036^*^

* *p* < 0.05

##### L2 acquisition, L1 attrition relationship

**Figure 3.**
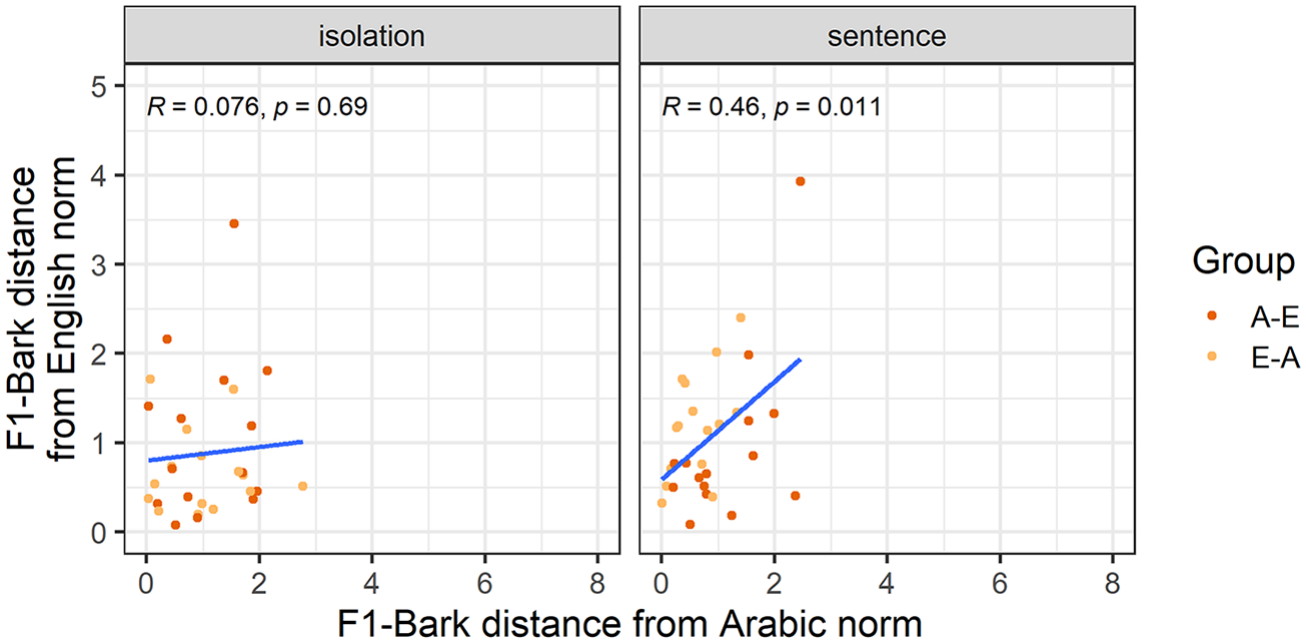
F1-Bark distance from the Arabic and English norms for the vowel /a-æ/.

**Figure 4.**
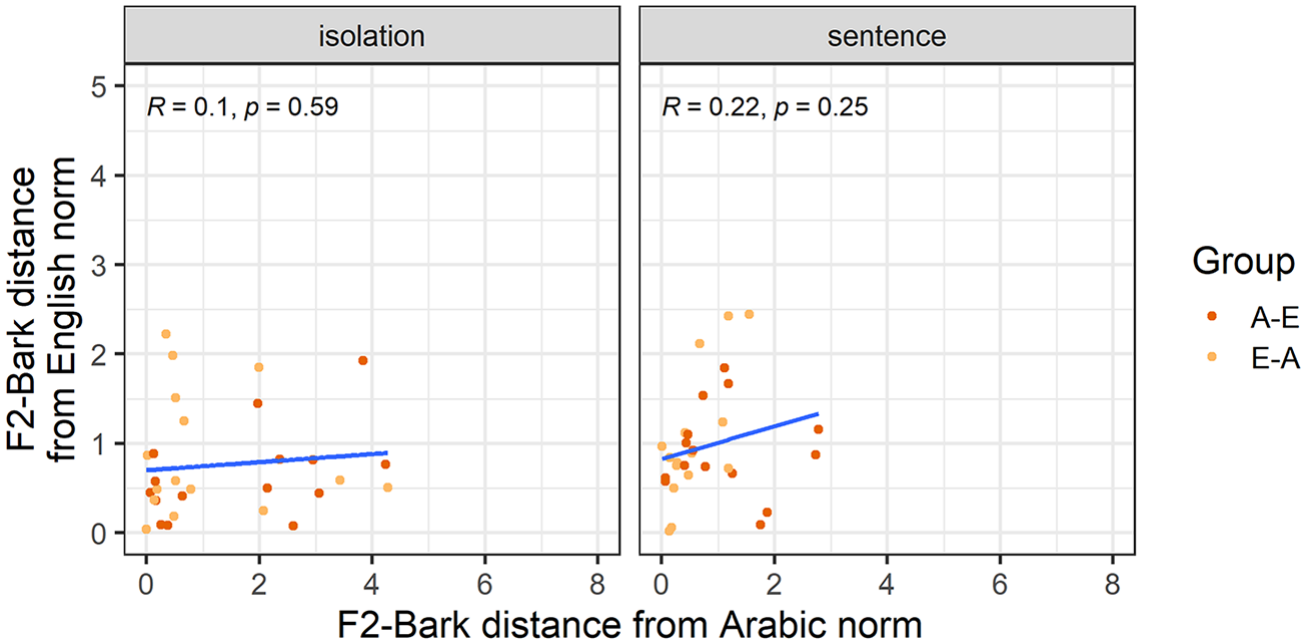
F2-Bark distance from the Arabic and English norms for the vowel /a-æ/.

Our third analysis explores whether nativelike realizations of L2 vowels entail nonnative productions of the corresponding L1 vowels. Figures 3 and 4 show how far each individual participant’s F1-Bark and F2-Bark are from monolingual Arabic and English norms. While all correlations had a positive correlation coefficient, we found a significantly positive correlation only for F1-Bark in the sentence condition, suggesting that for /a-æ/ in a carrier phrase, nativelike L2 realizations entail nativelike L1 productions in terms of tongue height.

##### /a/-/æ/ summary

Overall, our analyses suggests that the tongue is in a higher position for Arabic /a/ compared with English /æ/ and for the sentence condition compared with the isolation condition. The absence of any effects involving speaker group suggests that bilinguals have nativelike /a/-/æ/ productions in their respective L2s (RQ1) and show no L1 attrition (RQ2). We also found more target-like productions in terms of tongue frontness for English /æ/ than Arabic /a/ (RQ3). Finally, we found that, for the sentence condition, a more nativelike tongue height in the L2 relates to a more nativelike tongue height in the L1 (RQ4).

#### 3.2.2 Vowel /ɪ/

##### Experimental manipulations

**Figure 5.**
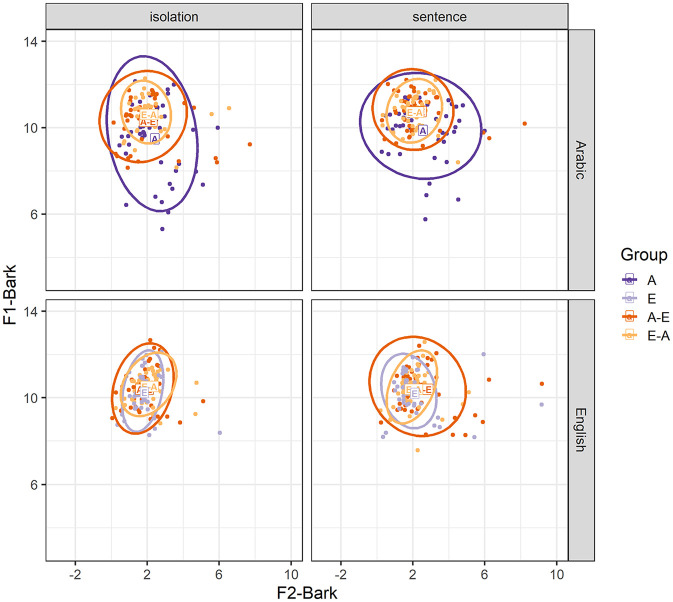
F1-Bark and F2-Bark values for Arabic and English /ɪ/ in the isolation and sentence conditions. The rectangles represent average F1-Bark and F2-Bark values for the Arabic monolinguals (A), English monolinguals (E), Arabic-English bilinguals (A-E), and English-Arabic bilinguals (E-A). Dots and ellipses show individual data points and distributions, respectively, for each participant group.

We now consider whether our experimental manipulations affect the F1-Bark and F2-Bark values of the vowel /ɪ/. Figure 5 reveals that for tongue height, Arabic /ɪ/ shows a larger spread of data points, particularly for monolingual Arabic productions, compared with English /ɪ/. In addition, Arabic /ɪ/ is produced at an overall higher position compared with English /ɪ/.

The final statistical model for F1-Bark (fixed effects: language, speaker group, speaking condition, speaker group by language interaction, speaking condition by language interaction; random intercepts: participants; random slopes: speaking condition by participant) in Table 3 shows a significant main effect of speaker group, but not of speaking condition and language. The analysis also revealed significant interactions for speaker group by language and speaking condition by language.

**Table 3.** Statistical results for the experimental manipulations analysis for F1-Bark for the vowel /ɪ/.

final statistical model				
*effect*	*β*	*SE*	*t*	*p*
speaker group	0.26	0.09	2.77	**=** 0.008^**^
speaking condition	0.06	0.04	1.44	= 0.157
language	-0.01	0.05	-0.30	= 0.761
speaker group by language	-0.10	0.05	-2.07	**=** 0.040^*^
speaking condition by language	-0.09	0.04	-2.51	**=** **0.012** ^*^
post-hoc simple contrasts: main effect of speaker group				
*comparison*	*β*	*SE*	*t*	*p*
mono vs. A-E biling	-0.52	0.23	-2.21	= 0.078
mono vs. E-A biling	-0.59	0.23	-2.54	**=** 0.037^*^
A-E biling vs. E-A biling	-0.08	0.27	-0.29	= 0.956
post-hoc simple contrasts: speaker group by language interaction				
*comparison*	*β*	*SE*	*t*	*p*
Arabic: mono vs. A-E biling	-0.85	0.28	-3.07	**=** 0.009^**^
Arabic: mono vs. E-A biling	-0.97	0.28	-3.51	**=** 0.002^**^
Arabic: A-E biling vs. E-A biling	-0.12	0.28	-0.45	= 0.896
English: mono vs. A-E biling	-0.18	0.28	-0.66	= 0.789
English: mono vs. E-A biling	-0.21	0.28	-0.76	= 0.728
English: A-E biling vs. E-A biling	-0.03	0.28	-0.10	= 0.994
mono: Arabic vs. English	-0.54	0.28	-1.94	= 0.057
A-E biling: Arabic vs. English	0.13	0.11	1.17	= 0.243
E-A biling: Arabic vs. English	0.23	0.12	1.98	**=** 0.049^**^
post-hoc simple contrasts: speaking condition by language interaction				
*comparison*	*β*	*SE*	*t*	*p*
Arabic: isolation vs. sentence	-0.31	0.11	-2.69	**=** 0.008^**^
English: isolation vs. sentence	0.05	0.11	0.46	= 0.647
isolation: Arabic vs. English	-0.24	0.13	-1.83	= 0.068
sentence: Arabic vs. English	0.12	0.13	0.97	= 0.334

* *p* < 0.05; **p < 0.01

For speaker group, post hoc comparisons showed only that the E-A bilinguals’ F1-Bark values are significantly higher compared with monolinguals.

For the group by language interaction, post hoc simple contrasts for speaker group revealed significant differences only for Arabic /ɪ/, with significantly higher F1-Bark values for both bilingual groups compared with monolinguals. The simple contrasts for language only revealed significant differences for E-A bilinguals, with significantly higher F1-Bark values for Arabic compared with English.

For the speaking condition by language interaction, post hoc simple contrasts for speaking condition revealed a significant difference only for Arabic /ɪ/, with significantly higher F1-Bark for the sentence than the isolation condition. The simple contrasts for language revealed no significant differences.

The final statistical model for F2-Bark (fixed effects: speaking condition; random intercepts: participants; random slopes: speaking condition by participant) in Table 4 shows a significant main effect of speaking condition, with significantly higher F2-Bark for /ɪ/ in the sentence condition compared with the isolation condition.

**Table 4.** Statistical results for the experimental manipulations analysis for F2-Bark for the vowel /ɪ/.

final statistical model				
*effect*	*β*	*SE*	*t*	*p*
speaking condition	0.11	0.06	2.02	**=** 0.048^*^

* *p* < 0.05

##### Individual differences

We now investigate if our individual differences measure affects the nativeness of bilinguals’ /ɪ/ productions. The final model for the F1-Bark distance-from-norm analysis (fixed effects: language; random intercepts: participants; random slopes: language by participant) in Table 5 shows a significant main effect for language, such that bilinguals’ productions are significantly closer to the monolingual norm in terms of tongue height for English /ɪ/ than for Arabic /ɪ/. We found no significant effect of sound discrimination aptitude on the nativeness of /ɪ/ in terms of tongue height.

**Table 5.** Statistical results for the individual differences analysis for F1-Bark for the vowel /ɪ/.

final statistical model				
*effect*	*β*	*SE*	*t*	*p*
language	-0.17	0.04	-3.81	**=** 0.001^**^

** *p* < 0.01

The final model for the F2-Bark distance-from-norm analysis had no fixed effects (random intercepts: participants; random slopes: language by participant). Sound discrimination aptitude did not affect the nativeness in terms of tongue frontness of our bilinguals’ /ɪ/ productions.

##### L2 acquisition, L1 attrition relationship

**Figure 6.**
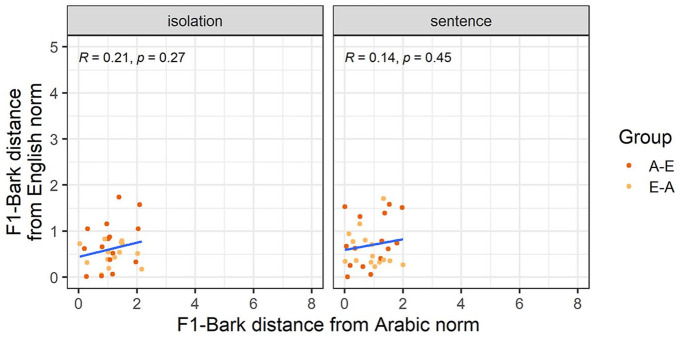
F1-Bark distance from the Arabic and English norms for the vowel /ɪ/.

**Figure 7.**
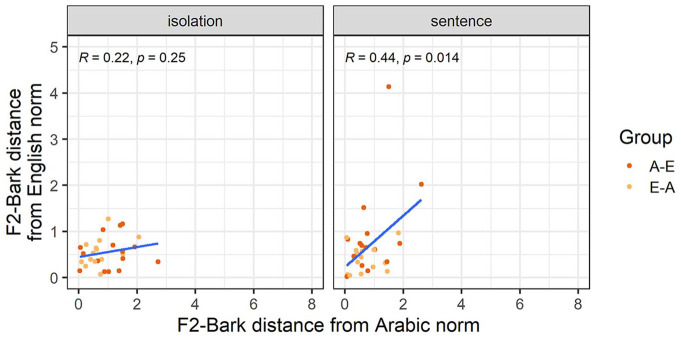
F2-Bark distance from the Arabic and English norms for the vowel /ɪ/.

The third analysis focuses on the acquisition-attrition relationship of the /ɪ/-vowel. Figures 6 and 7 show how far each participant’s F1-Bark and F2-Bark are from the monolingual Arabic and English norms, respectively. While all correlations again had a positive correlation coefficient, we found a significantly positive correlation only for F2-Bark in the sentence condition, suggesting that for /ɪ/ in a carrier phrase, nativelike L2 realizations entail nativelike L1 productions in terms of tongue frontness.

##### /ɪ/ summary

Overall, our analysis suggests that Arabic /ɪ/ is produced with a higher tongue position compared with English /ɪ/. Furthermore, E-A bilinguals produce Arabic /ɪ/ at an overall higher position than Arabic monolinguals, suggesting nonnative L2 productions (RQ1). Similarly, the A-E bilinguals produce Arabic /ɪ/ at a higher position than Arabic monolinguals, indicating L1 attrition (RQ2). No such effects were found for the E-A and A-E bilinguals’ English /ɪ/, suggesting nativelike productions (successful acquisition, no attrition). We also found more target-like productions in terms of tongue height for English compared with Arabic /ɪ/ (RQ3). Finally, we found that, for the sentence condition, a more nativelike tongue frontness in the L2 relates to a more nativelike tongue frontness in the L1 (RQ4).

#### 3.2.3 Vowel /u/-/ʊ/

##### Experimental manipulations

**Figure 8.**
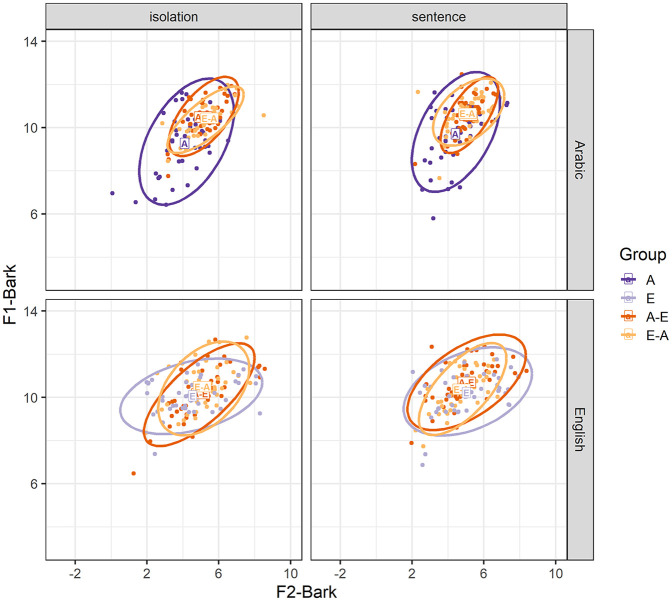
F1-Bark and F2-Bark values for Arabic /u/ and English /ʊ/ in the isolation and sentence conditions. The rectangles represent average F1-Bark and F2-Bark values for the Arabic monolinguals (A), English monolinguals (E), Arabic-English bilinguals (A-E), and English-Arabic bilinguals (E-A). Dots and ellipses show individual data points and distributions, respectively, for each participant group.

We first analyzed whether our experimental manipulations affect F1-Bark and F2-Bark values for /u-ʊ/. Figure 8 shows a larger spread of data points on both axes for Arabic /u/ compared with English /ʊ/. Furthermore, the bilinguals’ Arabic /u/ productions have overall higher F1-Bark and F2-Bark values, that is, a higher and more back position compared with the monolinguals.

The final statistical model for F1-Bark (fixed effects: speaker group, speaking condition, language, group by language interaction; random intercepts: participants) in Table 6 only shows significant main effects for speaker group and speaking condition, with the latter showing higher F1-Bark values for the sentence compared with the isolation condition. Post hoc pairwise comparisons for speaker group revealed that the two bilingual groups’ F1-Bark values were significantly higher than the monolinguals’ values, but the two bilingual groups did not differ significantly.

**Table 6.** Statistical results for the experimental manipulations analysis for F1-Bark for the vowel /u/-/ʊ/.

final statistical model				
*effect*	*β*	*SE*	*t*	*p*
speaker group	0.30	0.09	3.31	**=** 0.002^**^
speaking condition	0.11	0.04	3.04	**=** 0.002^**^
language	0.03	0.05	0.54	= 0.589
speaker group by language	-0.10	0.05	-1.96	= 0.051
post-hoc simple contrasts: main effect of speaker group				
*comparison*	*β*	*SE*	*t*	*p*
mono vs. A-E biling	-0.67	0.22	-3.08	**=** 0.009^**^
mono vs. E-A biling	-0.68	0.22	-3.11	**=** 0.008^**^
A-E biling vs. E-A biling	-0.01	0.25	-0.03	= 1.000

** *p* < 0.01

The final model for F2-Bark (fixed effects: speaker group, speaking condition, language, speaker group by speaking condition interaction, speaker group by language interaction; random intercepts: participants; random slopes: speaking condition by participant) in Table 7 shows a significant main effect of speaker group (but with no significant differences across groups in the post hoc tests) and significant interactions of speaker group by speaking condition and speaker group by language.

**Table 7.** Statistical results for the experimental manipulations analysis for F2-Bark for the vowel /u/-/ʊ/.

final statistical model				
*effect*	β	*SE*	*t*	*p*
speaker group	0.26	0.13	2.03	**=** **0.047** ^*^
speaking condition	0.01	0.05	0.18	= 0.860
language	-0.03	0.06	-0.50	= 0.617
speaker group by speaking condition	-0.16	0.05	-3.22	**=** 0.002^**^
speaker group by language	-0.15	0.06	-2.37	**=** 0.018^*^
post-hoc simple contrasts: main effect of speaker group				
*comparison*	β	*SE*	*t*	*p*
mono vs. A-E biling	-0.56	0.32	-1.73	= 0.202
mono vs. E-A biling	-0.60	0.32	-1.86	= 0.161
A-E biling vs. E-A biling	-0.04	0.37	-0.11	= 0.994
post-hoc simple contrasts: speaker group by speaking condition interaction				
*comparison*	β	*SE*	*t*	*p*
isolation: mono vs. A-E biling	-0.74	0.37	-2.01	= 0.119
isolation: mono vs. E-A biling	-0.99	0.37	-2.69	**=** 0.025^*^
isolation: A-E biling vs. E-A biling	-0.25	0.42	-0.60	= 0.822
sentence: mono vs. A-E biling	-0.37	0.32	-1.17	= 0.476
sentence: mono vs. E-A biling	-0.20	0.32	-0.64	= 0.800
sentence: A-E biling vs. E-A biling	0.17	0.36	0.47	= 0.885
mono: isolation vs. sentence	-0.40	0.16	-2.46	**=** 0.016^*^
A-E biling: isolation vs. sentence	-0.04	0.19	-0.19	= 0.852
E-A biling: isolation vs. sentence	0.39	0.19	2.06	**=** 0.047^*^
post-hoc simple contrasts: speaker group by language interaction				
*comparison*	β	*SE*	*t*	*p*
Arabic: mono vs. A-E biling	-0.90	0.38	-2.37	= 0.054
Arabic: mono vs. E-A biling	-1.07	0.38	-2.83	**=** 0.017^*^
Arabic: A-E biling vs. E-A biling	-0.17	0.38	-0.46	= 0.891
English: mono vs. A-E biling	-0.22	0.38	-0.58	= 0.833
English: mono vs. E-A biling	-0.12	0.38	-0.33	= 0.943
English: A-E biling vs. E-A biling	0.09	0.38	0.25	= 0.967
mono: Arabic vs. English	-0.56	0.37	-1.49	= 0.142
A-E biling: Arabic vs. English	0.13	0.14	0.90	= 0.372
E-A biling: Arabic vs. English	0.39	0.14	2.83	**=** 0.005^**^

* *p* < 0.05; **p < 0.01

For the speaker group by speaking condition interaction, post hoc simple contrasts for speaking condition showed only significantly higher /u/-/ʊ/ F2-Bark values for E-A bilinguals than monolinguals in the isolation speaking condition. Post hoc simple contrasts for speaker group further showed significantly higher /u-ʊ/ F2-Bark values for the sentence compared with isolation condition for monolinguals, and significantly lower /u-ʊ/ F2-Bark values for the sentence compared with isolation condition for E-A bilinguals.

For the speaker group by language interaction, post hoc simple contrasts for language only showed significantly higher F2-Bark values for E-A bilinguals compared with monolinguals for Arabic /u/. Post hoc simple contrasts for speaker group showed only that the E-A bilinguals’ F2-Bark values are significantly higher for Arabic /u/ than English /ʊ/.

##### Individual differences

We now analyze whether our individual differences measure affects the nativeness of bilinguals’ /u-ʊ/ productions. The final model for the F1-Bark distance-from-norm analysis (fixed effects: language; random intercepts: participants; random slopes: language by participant) in Table 8 shows a significant main effect of language, with more target-like productions for English than Arabic.

**Table 8.** Statistical results for the individual differences analysis for F1-Bark for the vowel /u/-/ʊ/.

final statistical model				
*effect*	*β*	*SE*	*t*	*p*
language	-0.17	0.06	-2.68	**=** 0.012^*^

* *p* < 0.05

The final model for the F2-Bark distance-from-norm analysis had no fixed effects (random intercepts: participants; random slopes: language by participant), such that there were no significant effects involving speaker group, language or sound discrimination aptitude.

##### L2 acquisition, L1 attrition relationship

**Figure 9.**
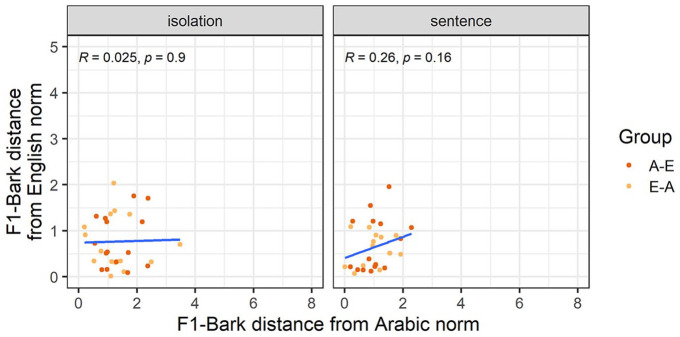
F1-Bark distance from the Arabic and English norms for the vowel /u-ʊ/.

**Figure 10.**
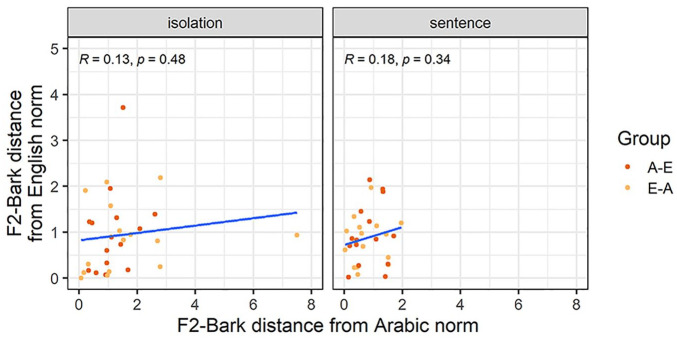
F2-Bark distance from the Arabic and English norms for the vowel /u-ʊ/.

The final analysis explores the acquisition-attrition relationship for /u-ʊ/. Figures 9 and 10 show how far each participants’ F1-Bark and F2-Bark are from the monolingual Arabic and English norms, respectively. While all Pearson correlations again had a positive correlation coefficient, none of them were significant. This suggests that, for the vowel /u-ʊ/, there is no direct relationship between successful L2 acquisition and L1 attrition/maintenance.

##### /u-ʊ/ summary

Overall, our analysis suggests that our bilinguals produced /u-ʊ/ at an overall higher position than the monolinguals in both languages. Furthermore, E-A bilinguals produced L2 /u/ with the tongue in a more back position (i.e., higher F2-Bark) than monolingual Arabic speakers, which suggests that they do not meet the native target for this L2 vowel (RQ1). At the same time, their L1 English /ʊ/ productions show no evidence of attrition (RQ2). No such effects were found for the A-E bilinguals, that is, both their L1 and L2 productions of /u-ʊ/ are nativelike. We also found more target-like productions in terms of tongue height for English /ʊ/ compared with Arabic /u/ (RQ3). Finally, we found no L2 acquisition, L1 attrition relationship for /u-ʊ/ (RQ4).

## 4 Discussion

The present study sought to identify whether long-term L2-immersed bilinguals produce the vowels /a-æ/, /ɪ/ and /u-ʊ/ with nativelike acoustic features in their L1 (Arabic/English) and L2 (English/Arabic) (RQ1, RQ2). In addition, we aimed to determine if and to what extent sound discrimination aptitude may account for individual differences in vowel production accuracy among our bilinguals (RQ3). A further objective was to gain insights into the relationship between L2 acquisition and L1 attrition of segmental productions in a bilingual context, that is, do nativelike L2 vowel productions entail attrition of the corresponding L1 vowels, or is the relationship between the two less straight-forward than that (RQ4)?

### 4.1 L2 acquisition (RQ1) and L1 attrition of vowels (RQ 2)

A number of acoustic studies has focused on the extent to which either only L1 (Bergmann et al., 2016; De Leeuw, 2019; Hévrová et al., 2020) or L1 *and* L2 (Kornder et al., 2023; Kornder & Mennen, 2021; Mayr et al., 2012) vowels of L2-immersed bilinguals are influenced by cross-language interactions. Although these studies differ in the number of vowels examined, there is a commonality in the results, which is also reflected in our findings: Even within the same segmental category, L1/L2 interactions seem to have different effects, that is, not all vowels are affected to the same extent, if at all. We made similar observations concerning our bilinguals’ plosive productions (see Alharbi et al., 2023), such that the bilinguals differed in the extent to which they were able to produce the target plosives in a nativelike fashion while at the same time maintaining nativelike production values for their L1 plosives.

Our vowel analysis revealed differences between the bilingual and the monolingual groups for the vowels /ɪ/ and /u-ʊ/: While producing English /ɪ/ with nativelike F1-Bark values (+ successful acquisition), the Arabic-English bilinguals realized the corresponding L1 vowel outside the monolingual Arabic norm (+ L1 attrition). The English-Arabic bilinguals, however, produced L2 Arabic /ɪ/ with nonnative F1-Bark values (–successful acquisition) while maintaining nativelike values for L1 /ɪ/ (–L1 attrition). Thus, only F1-Bark of Arabic /ɪ/ was affected. Similarly, we found higher F1-Bark for /u-ʊ/ in both bilingual groups compared with the monolinguals (–successful acquisition, +L1 attrition). As discussed by Mayr et al. (2012), changes affecting F1 are generally more likely to occur as the human ear is more sensitive to differences in lower than in higher frequencies, which could provide an explanation for our observations. We also found that F2-Bark of English /ʊ/ in an isolated condition was affected in one of the bilingual groups, such that the English-Arabic bilinguals produced L2 /u/ with F2-Bark-values outside the monolingual Arabic norm (–successful acquisition) while maintaining nativelike values in their L1 (–L1 attrition). The Arabic-English bilinguals, by contrast, produced both vowels in a nativelike fashion (+ successful acquisition, –L1attrition).

While differences between our bilingual and monolingual groups were observed for the vowels /ɪ/ and /u-ʊ/, the vowel /a-æ/ was produced within native ranges in both languages by both bilingual groups, that is, they successfully acquired the L2 vowel while maintaining nativelike values in their respective L1s. One reason for this may be found in the notion of similarity proposed by the SLM (Flege, 1995; Flege & Bohn, 2021). It posits that the greater the perceived distance between similar but acoustically nonidentical L1 and L2 sounds, the more likely it is that bilinguals establish two separate categories for these sounds. It might therefore be the case that the bilinguals in our study perceived Arabic /a/ and English /æ/ to be different enough—both acoustically and phonemically (Chang, 2019)—to categorize them as two distinct vowels.

The question of whether the two vowels are perceived to belong to the same or to different phonetic categories also raises the issue of how the relationship between perception and production accuracy should be characterized. Some models proposed in the context of L2 speech acquisition, such as the Perceptual Assimilation Model (PAM-L2; Best & Tyler, 2007), suggest that perception accuracy precedes production accuracy, that is, accurate perception is a prerequisite for accurate production. In contrast, more recent models, such as the Speech Learning Model-revised (SLM-r; Flege & Bohn, 2021) and ADAPPT (De Leeuw & Chang, 2024; see below for more details), argue that there is no clear link between speech perception and production. As noted by De Leeuw and Chang (2024), determining whether and to what extent production and perception accuracy are related requires a more comprehensive examination, not only of the acoustic-phonetic realizations of specific segmental and prosodic features, as investigated in the present study, but also of how individuals perceive these features. Therefore, the findings of our study do not allow for broad conclusions regarding the relationship between production and perception in bilinguals’ L1/L2. However, a vowel perception study could offer further insights into the extent to which our bilinguals perceive the Arabic and English short vowels as similar.

### 4.2 Individual differences (RQ3)

When it comes to how nativelike our bilinguals’ vowels were, we found that productions were overall more target-like for English than for Arabic. One possible explanation for this observation could be the widespread use of English across the world due to its role as a lingua franca. As a result of its international status, English is not only widely spoken—both as a native and even more so as a second or foreign language—but also frequently encountered in different media, and in educational and professional contexts. This widespread presence of English as a lingua franca may have contributed to a greater familiarity with its phonetic characteristics. This is likely to apply to both bilinguals whose L1 is Arabic but who live in an English-speaking country and to L1 English bilinguals.

Our predictor variable sound discrimination aptitude had no significant effects on the nativelikeness of /a-æ/, /ɪ/, and /u-ʊ/ productions. This finding may suggest that while language learning aptitude in general and phonetic aptitude in particular are often considered important factors in L2 acquisition (of pronunciation) (e.g., Baker Smemoe & Haslam, 2013), they may not be the ultimate determinant of production accuracy. Instead, external factors such as language exposure, quality and amount of L1/L2 input as well as the sociolinguistic status of Arabic and English, respectively, may either override the internal cognitive factor of aptitude or interact with it.

In summary, in terms of individual differences, we found more target-like productions for English compared with Arabic, potentially due to the role of English as a lingua franca. To gain a better understanding of how language learning aptitude may influence L2 acquisition and L1 maintenance, future studies need to more closely explore other cognitive skills associated with language learning aptitude, such as grammatical sensitivity, memory capacity or associative memory (i.e., the ability to remember new words) (see Carroll, 1981). Furthermore, future research should investigate the influence of sociolinguistic factors, such as language prestige, to provide a more comprehensive understanding of bilingual language acquisition and maintenance.

### 4.3 Relationship between acquisition and attrition (RQ4)

When it comes to characterizing the acquisition-attrition relationship in pronunciation, one hypothesis is that an increased L2 proficiency is likely to cause L1 proficiency to decline (e.g., Flege, 1987; Major, 1992). The relatively sparse number of studies (Alharbi et al., 2023; Kornder et al., 2023; Kornder & Mennen, 2021; Mayr et al., 2012; Stoehr et al., 2017) that examined the acquisition-attrition relationship in late bilinguals’ segmental productions supports this hypothesis, at least to some extent. Kornder et al. (2023), for example, found that the majority of their English-Austrian German bilinguals—despite some inter-speaker variability—produced the /i:/-/ɪ/ contrast in their L2 in a nativelike fashion, but at the same time exhibited nonnative productions of the corresponding L1 contrast. That is, their L1 vowel categories have approached L2-norms, presumably as a result of category assimilation (Flege, 1995; Flege & Bohn, 2021; see also Kornder & Mennen, 2021).

In our study, however, we observed quite the contrary. For the vowel /a-æ/ produced in a sentence, the closer our bilinguals’ F1-Bark Arabic values were to the monolingual Arabic norm, the closer were their English F1-Bark values to the monolingual English norm. We found the same significant positive correlation for the vowel /ɪ/ produced in a sentence, but only for F2. These findings suggest that bilinguals who produce L2 vowels in a nativelike fashion also maintain nativelike L1 categories. It may therefore be the case that nativelike categories in *both* languages can be established and maintained under certain circumstances, possibly also in response to additional factors, such as years of L2-immersion/experience, linguistic input, and age at first exposure to the L2. Similar observations were made by Mennen (2004) in a study with five participants, where the speaker who was most nativelike in their L2 values for pitch peak alignment also exhibited nativelike L1 values.

Adopting the SLM as a theoretical framework to contextualize our findings, the observation that some of our bilinguals exhibit nativelike vowel categories in both the L1 and the L2 may be explained against the background of the category precision hypothesis proposed in the revised version of the SLM (Flege & Bohn, 2021). According to this hypothesis, L2 learners’ ability to establish a new phonetic category for an L2 sound depends, among other factors, on how precisely the closest L1 category has been specified at the onset of L2 learning. That is, speakers whose L1 categories are relatively precise when L2 learning begins are assumed to more successfully distinguish an L2 sound from its closest L1 counterpart compared with bilinguals whose L1 categories are less precise. Precision in this context is defined as “the variability of acoustic dimensions measured in multiple productions of a phonetic category” (Flege & Bohn, 2021, p. 36). Consequently, precise *vowel* categories are those that display only little within-vowel variability, that is, are relatively more “compact” (Kartushina & Frauenfelder, 2013). To gain insights into whether L1 category precision affects L2 vowel production in our bilingual speaker groups, we would need to more closely examine vowel compactness and variability in the F1/F2 vowel space, perhaps alongside considering other acoustic features, such as vowel duration.

Taken together, the previous and present findings suggest that the acquisition-attrition relationship may change over the course of bilingual experience and is not as stable and straightforward as one may expect. Rather, it seems that this relationship is adaptive and highly variable, which is in line with a dynamic systems approach (DST) to language development (e.g., De Bot et al., 2007, 2013). According to DST, language development proceeds dynamically and in a nonlinear fashion, such that even small variations in one part of the system may lead to differences in outcome (De Bot et al., 2007). It may be due to such subtle system-internal modifications that, as our findings reveal, not all vowels show a significant positive L2 acquisition, L1 attrition relationship (see also Kornder & Mennen, 2021; Mayr et al., 2012). As proposed by DST, language systems constantly adjust not only to system-internal mechanisms (e.g., cross-linguistic interaction between the L1 and L2) but also to external factors (De Bot et al., 2007). Consequently, as stated above, an exploration of how motivational, attitudinal and emotional factors potentially influence nativelikeness of L1/L2 segmental speech production will shed further light on the relationship between acquisition and attrition in the phonetic domain.

The notion that language development is inherently dynamic is also reflected in one of the core principles of a more recent theory proposed by De Leeuw and Chang (2024) in the context of L1 attrition and drift. The *Attrition and Drift in Access, Production, and Perception Theory* (ADAPPT) posits, among other things, that there is no final stage of language development. Instead, the language systems of a bilingual speaker undergo continuous change throughout their lifespan, which is an inevitable consequence of the interaction between an individual’s language systems. As discussed earlier, our findings seem to support this assumption. However, understanding how the relationship between L2 acquisition and L1 maintenance/decline evolves over time requires not only cross-sectional studies but also long-term research. Even though the number of studies examining L1 attrition in bilingual pronunciation is steadily increasing (e.g., Alharbi et al., 2023; Bergmann et al., 2016; Dmitrieva et al., 2010; Mennen et al., 2022; Stoehr et al., 2017), there remains a great need for longitudinal studies (see De Leeuw, 2019; Kornder & Mennen, 2021, for exceptions) that trace various aspects of L1/L2 pronunciation development and assess the influence of potential predictor variables over an extended period of time.

## Figures and Tables

**Appendix B.**
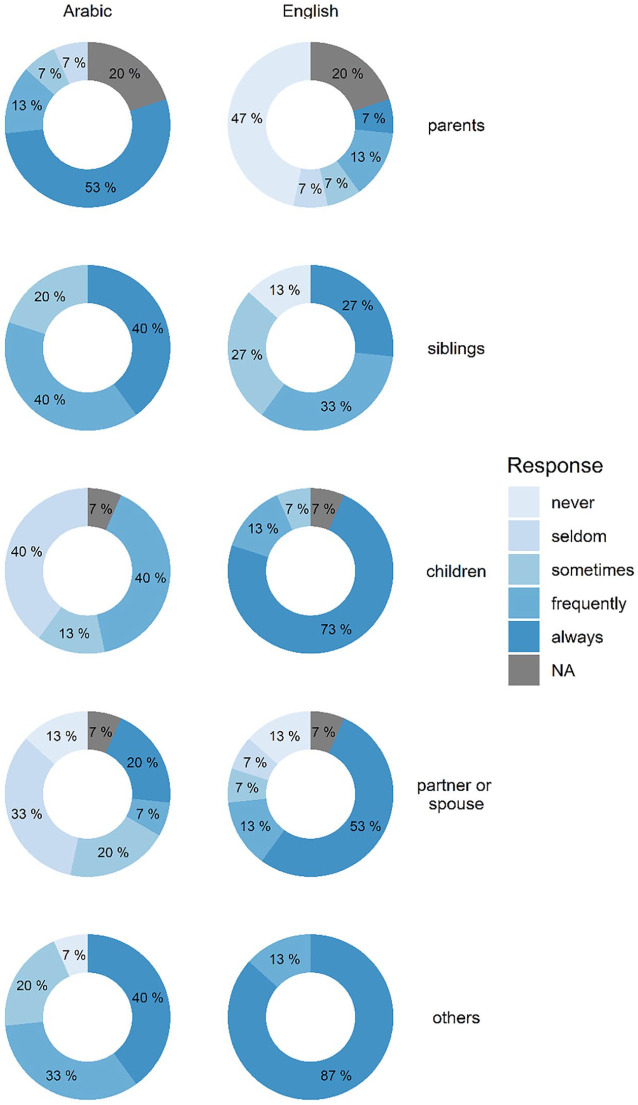
A-E bilinguals’ self-reported frequency of L1 (Arabic) and L2 (English) use with different people.

**Appendix C.**
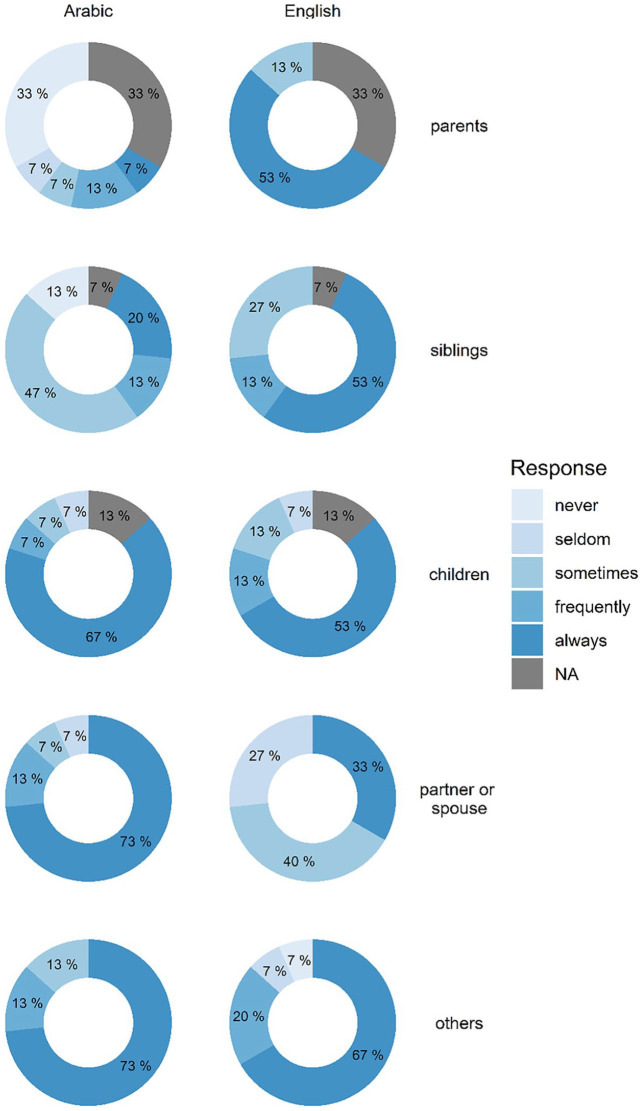
E-A bilinguals’ self-reported frequency of L1 (English) and L2 (Arabic) use with different people.

## Appendix

**Appendix A.** Examples of Arabic and English target words in isolated and sentence (embedded) conditions.

Arabic		English	
Isolation	Sentence	Isolation	Sentence
**qud, sˤud, hud***	qultu **qud** θumæ ðæhbt. qultu **sˤud** θʊmæ ðæhbt. qultu **hud** θumæ ðæhbt.	**would**, **could**, **hood**	I said **would** and then I left. I said **could** and then I left. I said **hood** and then I left.
“drive, block, hud*”	“I said drive, then I left. I said block, then I left. I said hud*, then I left.”		

**Appendix D.** F1 and F2 values in both Hertz and Bark for the vowel /a/-/æ/ for each speaker group, speaking condition, and language.

speaker group	speaking condition	formant	Arabic (Hertz)	English (Hertz)	Arabic (Bark)	English (Bark)
monolinguals	isolation	F1	703.58	805.44	8.34	7.63
		F2	1657.00	1563.83	3.20	3.55
	sentence	F1	631.20	797.68	8.85	7.78
		F2	1634.19	1493.44	3.20	3.94
A-E bilinguals	isolation	F1	725.71	845.88	8.10	7.13
		F2	1599.21	1640.17	3.47	3.03
	sentence	F1	696.26	873.43	8.52	7.25
		F2	1687.54	1660.12	3.23	3.27
E-A bilinguals	isolation	F1	710.91	820.39	8.10	7.61
		F2	1599.04	1576.9	3.34	3.59
	sentence	F1	696.16	794.12	8.64	7.62
		F2	1760.30	1603.98	3.03	3.27

**Appendix E.** F1 and F2 values in both Hertz and Bark for the vowel /ɪ/ for each speaker group, speaking condition, and language.

speaker group	speaking condition	formant	Arabic (Hertz)	English (Hertz)	Arabic (Bark)	English (Bark)
monolinguals	isolation	F1	607.9	523.74	9.49	10.25
		F2	1950.34	2183.84	2.43	1.85
	sentence	F1	537.22	523.0	9.87	10.19
		F2	1887.24	2112.19	2.57	2.11
A-E bilinguals	isolation	F1	493.21	502.49	10.29	10.38
		F2	2079.64	2163.61	2.06	1.85
	sentence	F1	481.90	509.96	10.75	10.39
		F2	2123.63	1989.86	2.25	2.62
E-A bilinguals	isolation	F1	490.72	508.99	10.61	10.52
		F2	2128.94	2132.23	2.10	2.13
	sentence	F1	484.97	513.42	10.72	10.38
		F2	2132.60	2127.54	2.12	2.06

**Appendix F.** F1 and F2 values in both Hertz and Bark for the vowel /u/-/ʊ/ for each speaker group, speaking condition, and language.

speaker group	speaking condition	formant	Arabic (Hertz)	English (Hertz)	Arabic (Bark)	English (Bark)
monolinguals	isolation	F1	586.98	536.92	9.27	10.07
		F2	1435.59	1467.76	4.10	4.54
	sentence	F1	564.37	520.06	9.71	10.22
		F2	1411.25	1351.64	4.42	5.02
A-E bilinguals	isolation	F1	479.64	495.31	10.49	10.17
		F2	1294.5	1297.73	5.13	4.99
	sentence	F1	491.66	491.01	10.58	10.70
		F2	1326.62	1395.93	5.14	5.04
E-A bilinguals	isolation	F1	502.16	497.51	10.43	10.49
		F2	1256.2	1339.53	5.48	5.08
	sentence	F1	490.0	510.89	10.66	10.38
		F2	1357.89	1406.25	5.09	4.76

## Notes

1. It should be noted that L2-on-L1 influences are not restricted to advanced bilinguals residing in an L2-country, but have also been evidenced among beginner learners (e.g., Chang, 2012, 2013; Dmitrieva et al., 2020; Kartushina et al., 2016) or bilinguals who experience regular changes in their linguistic environment (e.g., Sancier & Fowler, 1997; Tobin et al., 2017). Such L2-induced modifications of L1 pronunciation features are referred to as phonetic drift and are most likely to represent temporary changes that might be partially (Chang, 2019) or fully (Kartushina & Martin, 2019) reversed. In our study, we do not look at temporary phonetic drift, but are concerned with sustained modifications of L1 pronunciation features experienced by long-term L2-immersed bilinguals with a late L2-learning onset, that is, *L1 attrition of speech* (for a broader discussion of the term attrition see Gallo et al., 2021).

2. For the short a-vowels and the short u-vowels, Arabic and English use different IPA symbols. That is, the short counterpart to the English BOOK vowel /ʊ/ is the Arabic short vowel /u/; for the English TRAP vowel /æ/, Arabic uses the IPA symbol /a/ (see, for example, Abou Haidar, 1994; Al-Ani, 1970; Alghamdi, 1998; Alotaibi & Husain, 2009). We display these in pairs, showing the IPA character for the Arabic vowel first.

3. Due to the limited availability of participants fulfilling the inclusion criteria in terms of AoA and LoR, it was not possible to include a balanced number of male and female speakers in the different participant groups. We could therefore also not focus on gender as a potential variable in L2 acquisition and L1 attrition of vowel production.

4. The principal investigator of this study is a speaker of MSA (and L2 Standard Southern British English) herself and was therefore able to judge if the speakers did, in fact, use MSA in the speech production tasks.

5. The Arabic proficiency test was provided by the Language Institute of Umm Al-Qura University in Makkah Al-Mukarramah (Saudi Arabia). It is a placement test that students who want to study Arabic Language Teaching at Umm Al-Qura University have to pass.

6. While it is increasingly common to analyze formant trajectories over time (multiple-point measurements; see, for example, Di Paolo et al., 2011), we used single mid-point measurements since the controlled /hVd/ context minimized coarticulatory effects and ensured relatively stable vowel realizations. In such contexts, mid-point measurements can be considered to reflect the steady-state acoustic target of the vowel.

7. We chose not to report separate correlation analyses for the two bilingual groups (Arabic-English and English-Arabic) due to the small sample size (*N* = 15 per group) and because we do not have different expectations for Arabic-English and English-Arabic bilinguals. Regardless of whether the bilingual groups are considered separately or together, we found only nonsignificant and significant positive correlations, but no significant negative correlations.

8. Alternatives were Abou Haidar (1994), which includes just one male Saudi speaker, and Alotaibi and Husain (2009), who present data from nine male adult speakers and one child of unknown age together.

9. Our study includes seven male and 38 female English speakers. For each vowel, we therefore calculated the weighted averages using the formula (F1-male*7 + F1-female*38)/45 for F1 and (F2-male*7 + F2-female*38)/45 for F2.
